# Clinical Features and Key Prognostic Indicators of Growth Hormone-Secreting Pituitary Adenomas: A Retrospective Study of 344 Cases

**DOI:** 10.3390/curroncol33060310

**Published:** 2026-05-27

**Authors:** Yu Zhang, Zenghua Mi, Hongyu Wu, Xin Ma, Long Xi, Zhijun Yang, Pinan Liu

**Affiliations:** 1 Department of Neurosurgery, Beijing Tiantan Hospital, Capital Medical University, Beijing 100070, China; 2 Beijing Key Laboratory of Central Nervous System Injury, Department of Neural Reconstruction, Beijing Neurosurgical Institute, Capital Medical University, Beijing 100070, China

**Keywords:** GHPA, risk factors, tumor recurrence, hormonal non-remission

## Abstract

Background: In growth hormone-secreting pituitary adenomas (GHPA), postoperative hormonal non-remission and tumor recurrence determine clinical prognosis. Notably, some patients exhibit persistent hormonal abnormalities despite intraoperative gross total resection (GTR). This study systematically analyzes the prognostic risk factors in GHPA. Methods: This study collected clinical information from 344 GHPA patients. Univariate and multivariate regression analyses were performed to screen independent risk factors for hormonal non-remission, tumor recurrence, and hormonal non-remission after GTR. Nomograms were established for predicting hormonal non-remission and 3-/5-year recurrence probabilities. Subgroup comparisons were performed to further analyze significant risk factors. Results: Multivariate Logistic regression identified recurrent cases (*p* = 0.009), preoperative GH > 40 ng/mL (*p* = 0.043), not GTR (*p* < 0.001), and Knosp grade IV (*p* = 0.004) as independent risk factors of postoperative hormonal non-remission. For patients who underwent GTR, the recurrent status (*p* = 0.016) and Knosp grade III/IV (*p* = 0.049/0.009) remained significant risk factors for hormonal non-remission. Multivariate Cox regression showed preoperative GH > 40 ng/mL (*p* = 0.002), hormonal non-remission (*p* = 0.037), Knosp grade IV (*p* = 0.025), and vision defect (*p* = 0.010) as independent risk factors for tumor recurrence. The nomograms had good discrimination and calibration, with test set AUCs of 0.828 for hormonal non-remission, 0.82 for 3-year recurrence, and 0.859 for 5-year recurrence. Subgroup analysis revealed that recurrent cases, compared to initial cases, had younger patient ages, higher Knosp grades, harder tumor textures, and generally higher Ki-67 indices (all *p* < 0.05). Further analysis demonstrated that, relative to Knosp grade III tumors, grade IV lesions were larger, firmer, and associated with significantly lower rates of both GTR and hormonal remission, alongside a higher recurrence risk (all *p* < 0.05). Conclusions: Patients with high preoperative GH levels, higher Knosp grades, and recurrence often have a poorer prognosis after treatment. These results support stratified postoperative management and individualized monitoring of GHPA patients.

## Simple Summary

Growth hormone-secreting pituitary adenomas are common functional pituitary tumors that cause acromegaly and serious complications. Surgery is the main treatment, but some patients still have abnormal hormone levels or tumor recurrence after total resection, and the related risk factors are not fully clear. This study analyzed 344 patients and identified recurrence, high preoperative growth hormone levels, and high Knosp grades as key risk factors for poor prognosis. We also built prediction models for hormone non-remission and recurrence risk. These results can help doctors stratify patient management, optimize follow-up plans, and improve the prognosis of patients with this tumor.

## 1. Introduction

Growth hormone-secreting pituitary adenoma (GHPA) accounts for approximately 12% of all pituitary adenomas [1] and is a clinically prevalent functional pituitary tumor. Excessive production of GH is the core characteristic of the disease, which can lead to acromegaly. Its clinical manifestations include acral enlargement, facial dysmorphism, cardiovascular complications, and reproductive dysfunction [2,3,4]. The clinical sequelae resulting from elevated GH levels severely impair patients’ quality of life. Currently, surgery constitutes the first-line treatment for GHPA [5], encompassing transcranial tumor resection and transnasal endoscopic tumor resection; among these, endoscopic surgery serves as the preferred approach, yielding superior postoperative hormonal control [6,7,8]. In addition to surgical resection, the clinical management of GHPA also includes medical interventions such as somatostatin analogs, dopamine agonists, and growth hormone receptor antagonists, which can be applied for preoperative preparation or postoperative adjuvant therapy according to individual clinical conditions. Postoperative, a subset of patients fails to achieve hormonal remission or even develops tumor recurrence. In addition, during clinical observation, we found that the postoperative hormone levels remained unremitted in some patients who achieved GTR intraoperatively.

Previous literature reports that the hormonal remission rate is approximately 60% [9,10,11]. Hormonal non-remission is associated with multiple factors such as the ki-67 index, Knosp grade, and preoperative GH levels [12,13]. Previous studies on GHPA diagnosis and treatment are often limited by small sample sizes or incomplete clinical indicators, hindering comprehensive comparative analyses of postoperative prognostic factors. Therefore, this study conducted a retrospective analysis of clinical data from patients with GHPA admitted to Beijing Tiantan Hospital. By employing a relatively large sample size and comprehensive clinical indicators, we conducted an in-depth analysis of risk factors associated with postoperative hormonal non-remission, tumor recurrence, and hormonal non-remission after GTR. Furthermore, we performed subgroup comparisons of significant risk factors to elucidate the differences in clinical characteristics between high-risk patients and non-high-risk patients. This study aims to provide evidence-based support for postoperative stratified management, long-term follow-up, and individualized monitoring of such patients.

## 2. Materials and Methods

**Figure 1:**
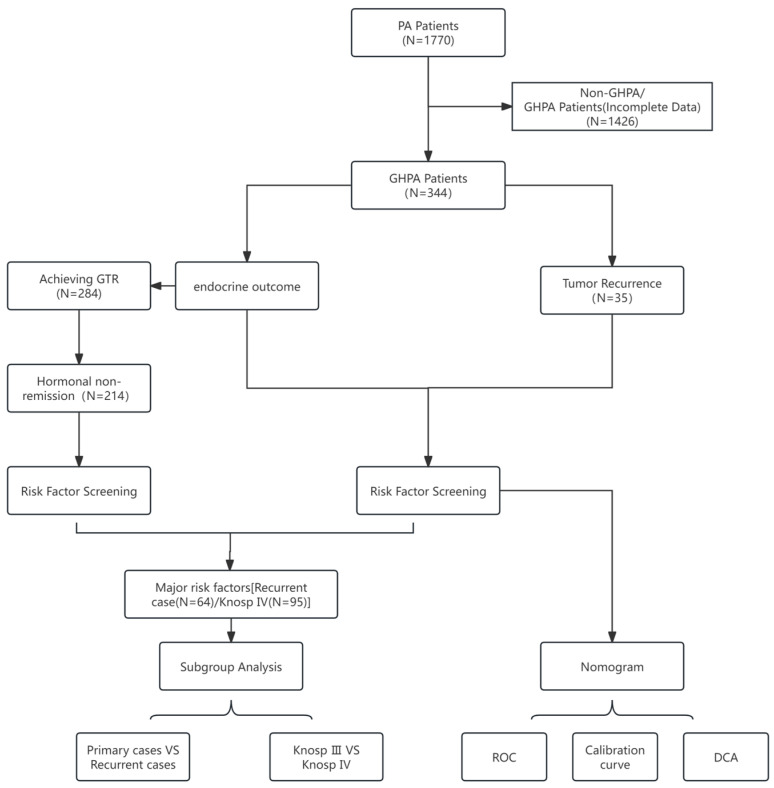
Workflow of this study.

We performed a retrospective cohort study of GHPA patients who underwent surgical treatment at Beijing Tiantan Hospital. The study was approved by the Ethics Committee of Beijing Tiantan Hospital, Capital Medical University (KY2025-007-02). Informed consent was obtained from all individual participants included in the study. Clinical data—including patients’ basic information, imaging findings, preoperative examination results, intraoperative details, and postoperative outcomes—were systematically extracted from the electronic medical record system and imaging database. The primary objective of this study was to identify the risk factors associated with GHPA prognosis. The workflow of this study is illustrated (Figure 1).

### 2.1. Patient Cohort

We retrospectively collected data from GHPA patients who underwent surgical treatment at Beijing Tiantan Hospital between January 2018 and February 2025. The inclusion criteria were as follows: (1) GHPA was confirmed by postoperative histopathological examination with immunohistochemistry and endocrinological assessment; (2) serum growth hormone (GH) levels exceeding the normal range or presence of acromegaly-related symptoms. The exclusion criteria were: (1) Incomplete clinical data (e.g., missing postoperative contrast-enhanced magnetic resonance imaging [MRI] scans); (2) discharge without surgical intervention after hospitalization.

### 2.2. Variable Selection

We collected comprehensive data from 344 GHPA patients who underwent surgical treatment at Beijing Tiantan Hospital between January 2018 and February 2025. The data encompassed patients’ basic characteristics (sex, age, body mass index [BMI], recurrence status, hypertension, and diabetes), disease-related manifestations (vision defect and headache), imaging findings (maximum tumor diameter, Knosp grade, pituitary apoplexy, pituitary cyst, and suprasellar extension), surgical details (tumor vascularity, intraoperative blood loss, GTR, tumor consistency, and intraoperative cerebrospinal fluid [CSF] leak), postoperative recovery parameters (postoperative infection, and length of hospital stay [LOS], follow-up time and laboratory indicators preoperative GH level, insulin-like growth factor-1 [IGF-1], postoperative hormonal remission status, and Ki-67 index). Patients’ recurrence status was collected during follow-up, and the time of recurrence was recorded if tumor recurrence occurs. GH was categorized as an ordinal variable (<20 ng/mL, 20–40 ng/mL, and >40 ng/mL), while the Ki-67 index was dichotomized (<3% and ≥3%). A complete dataset was available for most patients; those with incomplete data were excluded. All clinical evidence was evaluated by experienced researchers.

Vision defect is defined as the occurrence of decreased vision or visual field defects. Pituitary apoplexy is defined as acute hemorrhage and/or infarction of the pituitary gland. Pituitary cystic change refers to the development of fluid-filled cavities within the pituitary gland. The first postoperative follow-up MRI is performed 3 months after discharge, and subsequently once a year thereafter. Recurrence of GHPA is defined as the appearance of a new enhancing tumor focus on T1-weighted image, or further progression of residual tumor compared with previous imaging. GTR is defined as: intraoperative assessment by the surgeon confirming complete removal of all visible tumor, with no evidence of residual tumor on postoperative contrast-enhanced MRI.

### 2.3. Imaging Assessment

Magnetic resonance imaging (MRI) was utilized for preoperative assessment and postoperative follow-up. This study primarily focuses on T1-weighted images. All patients underwent preoperative contrast-enhanced MRI at our institution. Postoperative follow-up MRI was performed within 3 days after surgery to evaluate residual tumor, as well as during subsequent outpatient visits after discharge used to check whether tumor recurrence occurs. The maximum tumor diameter was calculated based on measurements from axial, coronal, and sagittal T1-weighted sequences of preoperative MRI. The achievement of GTR was determined by integrating postoperative MRI findings with surgical records. The Knosp grading system was adopted to evaluate cavernous sinus invasion of pituitary adenomas. This radiological classification is based on the spatial relationship between the tumor and internal carotid artery, and is widely used to assess tumor invasiveness clinically.

Knosp grading and whether the tumor recurs was independently assessed by two senior neurosurgeons in accordance with predefined criteria; in cases of discrepancies, reassessment was conducted until a consensus was reached.

### 2.4. Endocrine Diagnostic Criteria

According to guidelines from the Endocrine Society, Hormonal remission was defined as the normalization of IGF-1 levels adjusted for age and sex, a basal GH level < 1 ng/mL was regarded as an indicator of surgical remission; if basal GH levels exceeded 1 ng/mL, an oral glucose tolerance test (OGTT) was performed [2]. Remission was confirmed when the nadir GH level during OGTT reached <0.4 ng/mL [14,15]. Random GH (also referred to as basal GH) was measured in the morning following an overnight fast, with no glucose load. The 75 g OGTT was administered after an overnight fast, and serum GH levels were assessed at 0, 60, 90, 120, and 180 min post-administration. The nadir GH was defined as the lowest GH value detected during OGTT.

### 2.5. Statistical Analysis

Continuous variables following a normal distribution were expressed as mean ± standard deviation, while those deviating from normality were presented as median (interquartile range [IQR]). Categorical variables were described as frequency (percentage). For intergroup comparisons, the independent samples *t*-test or Mann–Whitney U test was used for continuous variables, and the Chi-square test or Fisher’s exact test for categorical variables. Variables with a *p*-value < 0.05 in univariate analysis were incorporated into multivariate regression models to identify independent risk factors for each outcome. Risk factors for hormonal non-remission were screened using logistic regression, while risk factors for tumor recurrence were screened using Cox regression. Based on the results of multivariate regression analyses, nomograms were constructed, with the study dataset partitioned into a training set and a validation set at a ratio of 7:3 to support the nomogram building process. The discriminative ability and calibration of the constructed nomograms were evaluated using receiver operating characteristic (ROC) curves, calibration curves and decision curve analysis (DCA), respectively. All statistical analyses were performed using R software (version 4.0.2), with *p* < 0.05 considered statistically significant.

## 3. Results

### 3.1. The Clinical Behaviors of Patients with GHPA

This study collected clinical information from 344 GHPA patients. During a median follow-up of 25 months (IQR: 12.0–39.0), 35 (10.2%) cases of recurrence were recorded. The cohort consisted of 54.9% (189/344) female and 45.1% (155/344) male patients, with a mean age of 41.76 ± 12.67 years. Recurrent cases accounted for 18.6% (64/344) of patients, while primary cases comprised 81.4% (280/344). The most common preoperative symptoms were vision defect in 29.7% (102/344) and headache in 28.2% (97/344); hypertension and diabetes were present in 20.3% (70/344) and 18.6% (64/344) of patients, respectively. Preoperatively, 64.8% (223/344) of patients had GH levels < 20 ng/mL, and the mean IGF-1/ULN ratio was 1.87 ± 0.80. Radiologically, the mean maximum tumor diameter was 22.13 ± 11.30 mm. Regarding Knosp grade, grade IV was the most frequent at 27.6% (95/344), followed by grade III at 19.8% (68/344), grade II at 19.2% (66/344), grade I at 19.2% (66/344), and grade 0 at 14.2% (49/344). Suprasellar extension was observed in 53.2% (183/344) of cases. Gross-total resection (GTR) was achieved in 82.6% (284/344) of patients; hypervascular tumors accounted for 64.2% (221/344), and the mean intraoperative blood loss was 263.95 ± 343.72 mL, the intraoperative blood loss ranged from a minimum of 20 mL to a maximum of 3500 mL. Additional detailed characteristics are summarized (Supplementary Table S1).

### 3.2. Persistence of Hormonal Overproduction

**Figure 2:**
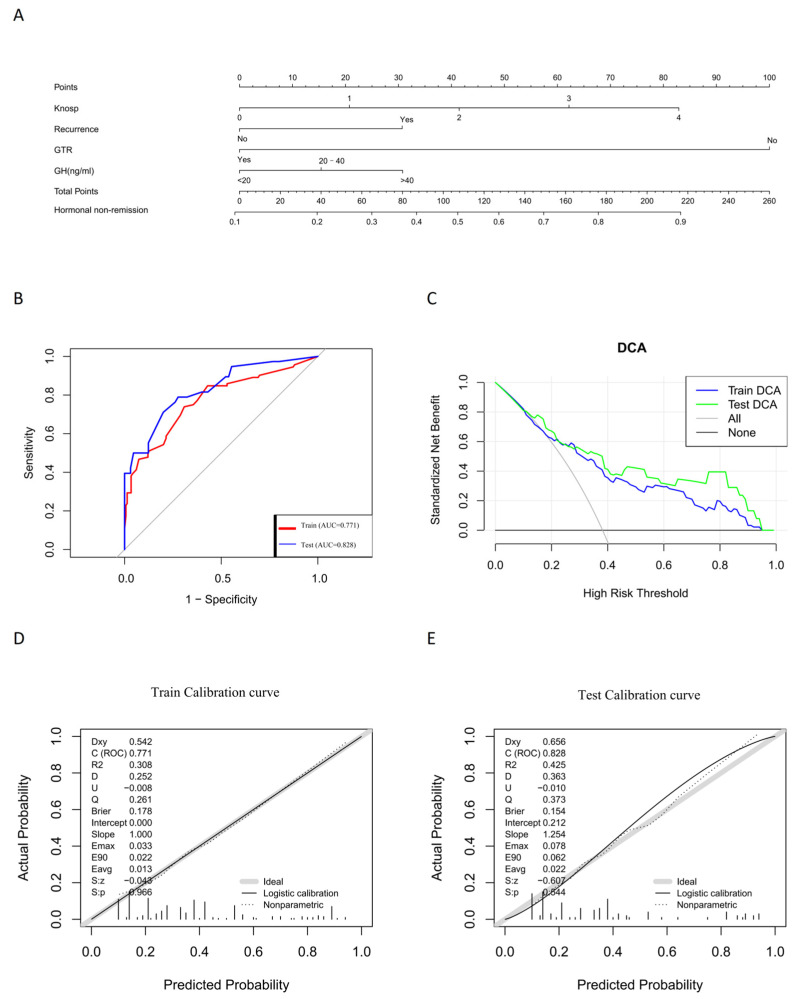
Nomogram, ROC curve, Calibration curves, and DCA based on Logistic regression prediction models: (**A**). Hormonal Non-remission’s Nomogram; (**B**). Hormonal Non-remission’s ROC; (**C**). Hormonal Non-remission’s DCA; (**D**). Hormonal Non-remission’s Train Calibration curve; (**E**). Hormonal Non-remission’s Test Calibration curve.

In this study, 214 (62.2%) patients achieved postoperative Hormonal remission. Univariate Logistic regression analysis revealed that age, recurrence status, preoperative GH level, maximum tumor diameter, Knosp grade, GTR, intraoperative blood loss, and tumor consistency were significantly associated with postoperative hormonal non-remission (all *p* < 0.05). These factors were further incorporated into multivariate Logistic regression analysis, which identified recurrent cases (*p* = 0.009), preoperative GH > 40 ng/mL (*p* = 0.043), not-GTR (*p* < 0.001), and Knosp grade IV (*p* = 0.004) as independent risk factors of postoperative Hormonal non-remission (Table 1). These independent risk factors were integrated into model development and nomogram construction (Figure 2A), with ROC curves (Figure 2B), calibration curves (Figure 2D,E) and DCAs (Figure 2C) generated to validate model performance.

### 3.3. Recurrence

**Figure 3:**
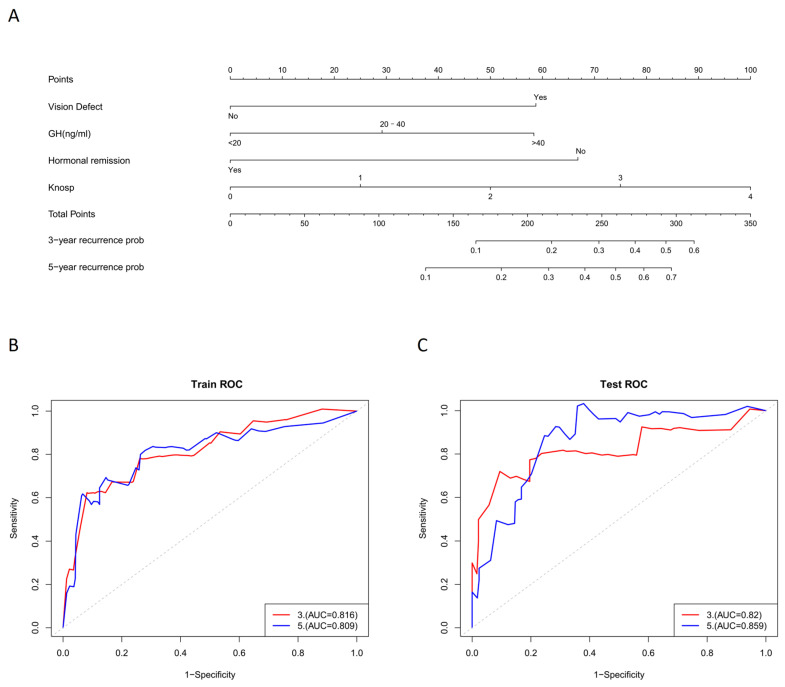
Nomogram and ROC curves based on COX regression prediction models: (**A**). Nomogram for Predicting Tumor Recurrence; (**B**). Training Set ROC for Recurrence Prediction; (**C**). Testing Set ROC for Recurrence Prediction.

**Figure 4:**
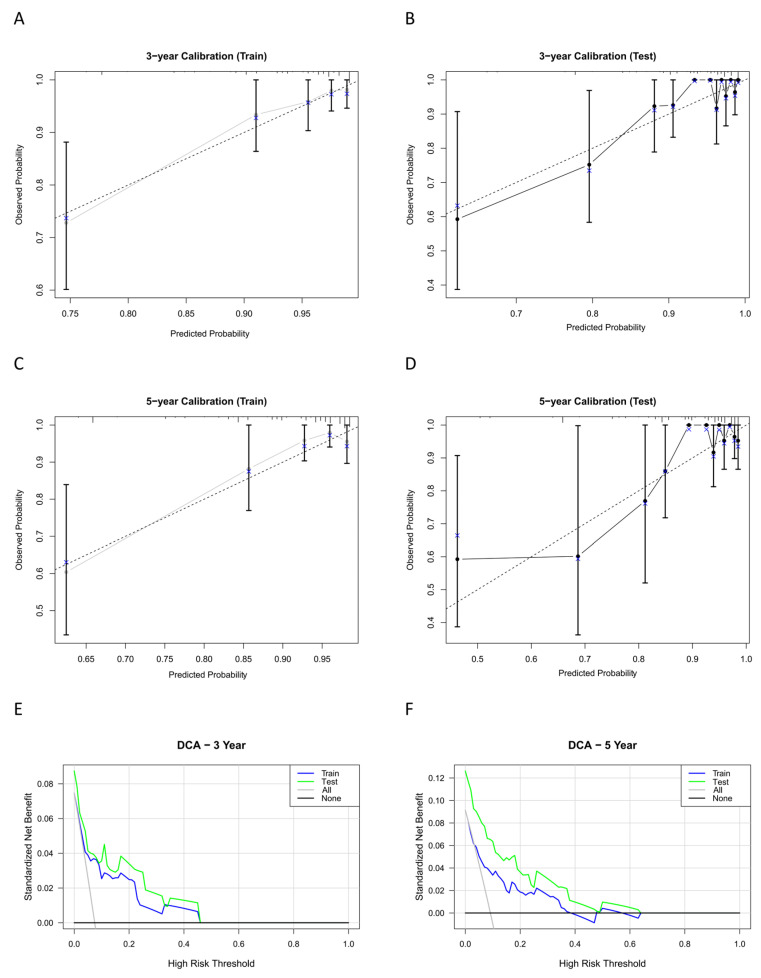
Calibration curves and DCAs based on COX regression prediction models (**A**). 3-year recurrence calibration curve (Training set); (**B**). 3-year recurrence calibration curve (Testing set); (**C**). 5-year recurrence calibration curve (Training set); (**D**). 5-year recurrence calibration curve (Testing set); (**E**). 3-year recurrence DCA; (**F**). 5-year recurrence DCA.

In this study, factors potentially associated with tumor recurrence were analyzed using clinical data from patients. Univariate analysis identified factors with statistically significant differences, including recurrence status, visual defect, preoperative GH level, maximum tumor diameter, Knosp grade, GTR, LOS, Ki-67 index, and postoperative hormonal non-remission. These factors were subsequently incorporated into multivariate COX regression analysis, which revealed that preoperative GH > 40 ng/mL, postoperative hormonal non-remission, Knosp grade IV, and preoperative visual defect (All *p* < 0.05) were independent risk factors for tumor recurrence (Table 2). These independent factors were further integrated into a Cox regression model to construct nomogram predicting 3-year and 5-year tumor recurrence probabilities (Figure 3A), with ROC curves (Figure 3B,C); calibration curves (Figure 4A–D); and DCAs (Figure 4E,F) generated for validation.

### 3.4. Hormonal Non-Remission After GTR

In this study, we observed that a subset of patients (N = 81) (28.5%) who achieved GTR intraoperatively still did not attain hormonal remission. To further investigate this, we analyzed risk factors associated with postoperative hormonal non-remission specifically in patients who underwent GTR (Table 3). Univariate analysis identified recurrent cases, maximum tumor diameter, and Knosp grade as factors with statistically significant differences (All *p* < 0.05). Subsequent multivariate analysis revealed that among patients who achieved GTR, independent risk factors for failure to achieve postoperative hormonal remission were recurrent tumor status (*p* = 0.016) and Knosp grade III/IV (Knosp III: *p* = 0.049/Knosp IV: *p* = 0.009). Therefore, we conclude that even with intraoperative GTR, patients with recurrent tumors or highly invasive tumors (Knosp III/IV) are less likely to achieve hormonal remission.

### 3.5. Subgroup Analysis of Risk Factors

In the study, we found that the prognosis of recurrent cases differs significantly from that of primary cases. To systematically characterize the differences between primary and recurrent tumors, patients were stratified into primary (N = 280) and recurrent (N = 64) cohorts for comparative analysis (Table 4). Significant differences were found in several key areas: patients in the recurrent group were younger (*p* = 0.015), presented with larger tumors (*p* = 0.016), had a higher incidence of visual defects (*p* = 0.002), and showed a more invasive Knosp grade distribution (*p* = 0.002), with a notable predominance of grade IV lesions. Intraoperatively, recurrent tumors were associated with greater blood loss (*p* = 0.024), a firmer consistency (*p* < 0.001), and a significantly lower rate of gross total resection (GTR) (*p* < 0.001). Pathologically, they exhibited a higher proliferative index, with more cases having a Ki-67 ≥ 3% (*p* < 0.001). Consequently, the postoperative GH remission rate was substantially lower in the recurrent group (*p* < 0.001). In summary, recurrent GH adenomas demonstrate a more aggressive clinicopathological profile, including larger size, greater invasiveness, and higher proliferative activity, which collectively contribute to greater surgical challenges and inferior endocrine outcomes.

Although both Knosp grade III and IV are considered invasive, our study indicates that grade IV tumors are associated with a poorer prognosis. Therefore, patients were further stratified into Knosp grade III (N = 68) and grade IV (N = 95) subgroups for comparative analysis (Table 4). The results revealed that, compared with grade III tumors, grade IV tumors had a significantly larger maximum diameter (*p* < 0.001), greater intraoperative blood loss (*p* < 0.001), and firmer consistency (*p* = 0.027). Correspondingly, the gross total resection (GTR) rate was significantly lower (*p* = 0.002) and the postoperative GH remission rate was worse (*p* = 0.003) in the grade IV subgroup. Furthermore, grade IV patients had significantly higher rates of postoperative infection (*p* = 0.011) and tumor recurrence (*p* = 0.011). No significant differences were observed between the two subgroups in terms of sex, age, BMI, comorbidities, GH and IGF-1 levels, or preoperative symptoms such as headache and vision defects.

## 4. Discussion

GHPA is a common functional pituitary adenoma. This study retrospectively analyzed prognostic factors. Hormonal non-remission and tumor recurrence are closely linked to patient outcomes. Notably, some patients fail to achieve hormonal remission despite GTR. We focused on risk factors for hormonal non-remission, tumor recurrence, and post-GTR non-remission. Results showed that tumor recurrence and higher Knosp grade (especially grade IV) are significant prognostic risk factors. Further comparative analyses were conducted between recurrent and primary cases, as well as within Knosp grades. While previous studies have primarily focused on the differences in invasiveness between Knosp grades 0/I/II and grades III/IV [16], our study found that patients with Knosp grade IV had a significantly poorer prognosis. Accordingly, this study specifically compared cases of Knosp grade III and grade IV to explore in detail the differences in clinical prognosis between these two subgroups.

Numerous studies have indicated that immediate postoperative hormonal remission is a strong predictor of long-term postoperative hormonal remission [10,17,18]. In the present study, GH levels measured on the first postoperative day were used as the indicator for hormonal remission. A large body of previous research has explored the risk factors associated with postoperative hormonal non-remission in GHPA patients, identifying preoperative GH level, Knosp grade, GTR, and recurrence status as closely relevant factors [19,20,21,22,23]. Previous literature classifies cases with Knosp grade III/IV as invasive [16]; however, our study suggests that Knosp grade IV is more crucial for predicting postoperative hormonal remission. Prior scholars have reported that Knosp grade IIIA and IIIB exert distinct impacts on prognosis, with grade IIIA associated with significantly better outcomes than IIIB [24,25]. Unlike Knosp grade IV, grade III tumors do not fully encircle the internal carotid artery. Even for experienced endoscopic neurosurgeons, resection of grade IV tumors remains challenging, with lower postoperative hormonal remission rates and poorer prognosis. In the following sections, we compare grade III and grade IV patients to examine differences in clinical characteristics.

Our previous research has also confirmed that preoperative GH level, GTR, and recurrence status are closely associated with patients’ postoperative hormonal non-remission [26]. In the present study, we integrated all potential influencing factors to evaluate the risk factors affecting postoperative hormonal non-remission by leveraging a larger sample size and more comprehensive clinical indicators. Among these factors, not-GTR was found to exert a significant impact on hormonal non-remission. Our prior work indicated that residual tumor substantially influences patient prognosis, and key factors affecting the resection rate include Knosp grade, tumor size, and whether the surgery is the first-time intervention [22]. Previous studies have identified recurrence status and tumor volume as closely relevant to GTR [27,28,29,30]. Recurrent cases are often more complex than primary cases, increasing surgical difficulty and making postoperative hormonal remission more challenging. Further subgroup analyses were subsequently conducted.

In the diagnosis and treatment of GHPA, tumor recurrence remains a persistent concern for patients. We conducted an analysis of potential risk factors associated with postoperative tumor recurrence. Previous studies have reported that preoperative hormone levels and Knosp grade are correlated with tumor recurrence [31,32,33], and our research yielded consistent findings. Among Knosp grades, grade IV tumors fully encircle the internal carotid artery, posing greater surgical challenges that may lead to residual tissue and recurrence. Postoperative hormonal non-remission also serves as a risk factor for recurrence. Conversely, hormonal remission strongly predicts long-term control, typically reflecting GTR and low tumor invasiveness, making recurrence less likely. In addition, for patients with residual or recurrent adenomas and persistent hormonal non-remission after surgery, radiosurgery can serve as an important supplementary treatment to control tumor progression and ameliorate endocrine disorders.

In our cohort, patients with preoperative optic chiasm compression symptoms were more prone to recurrence during postoperative follow-up. Vision Defect is a common manifestation in pituitary adenomas [34], and tumor compression of the optic chiasm often indicates poor prognosis [35,36] and low resection rates [37]. Such compression usually indicates suprasellar extension with anterior displacement, which increases surgical difficulty and recurrence risk. In contrast, preoperative confirmation of suprasellar extension did not show significant clinical significance in this study.

Notably, some patients who achieved intraoperative GTR still failed to attain postoperative hormonal remission. Our study revealed that in patients with Knosp grade III/IV pituitary adenomas and those with recurrent tumors, hormone levels often remain elevated even after GTR is achieved. This may be attributed to the more invasive nature typically associated with Knosp grade III/IV [16]. Comparative analysis between primary and recurrent cases showed a significant difference in tumor invasiveness (see detailed analysis below). Infiltrative growth, a hallmark of aggressive tumors, involves diffuse micro-invasion into adjacent structures such as the dura mater, cavernous sinus, bone, and perivascular or perineural spaces. These micro-invasive areas are difficult to delineate during surgery and often evade detection by conventional MRI and intraoperative visualization, making residual foci likely even after GTR. Additionally, aggressive tumors typically exhibit high cellular proliferative activity, meaning even a few residual cells can sustain elevated hormone secretion, leading to persistent postoperative hormonal elevation. Therefore, we propose that among patients who underwent GTR with similar baseline characteristics, those with highly invasive tumors are less likely to achieve postoperative hormonal remission.

With the advancement of endoscopic technology, transnasal endoscopic surgery has currently become the preferred treatment for GHPA patients [38]. Most recurrent GHPA patients admitted to our hospital had previously undergone transnasal endoscopic surgery, resulting in varying degrees of nasal cavity structural damage. In this study, patients were stratified into primary and recurrent subgroups for comparative analysis, revealing significant differences. The recurrent group showed a significantly higher Ki-67 index on postoperative immunohistochemistry and firmer tumor consistency intraoperatively, which increases resection difficulty during reoperation. Additionally, recurrent patients had larger tumor volumes, a higher proportion of visual defects, and a greater prevalence of Knosp grade IV tumors. This indicates that recurrent tumors are generally more invasive, significantly increase the difficulty of reoperation, and lead to poorer postoperative recovery [39]. Recurrent patients were younger, suggesting younger age may predict incomplete resection and poorer prognosis. Consistent with this, previous scholars have reported that younger patients tend to have more invasive tumors and worse prognosis compared to older counterparts [40].

Previous literature considered both Knosp grade III and Knosp grade IV to be indicative of invasive tumors with infiltrative growth patterns [16]. However, in recent years, some scholars have pointed out that there are significant differences between Knosp III and Knosp IV in terms of disease diagnosis and treatment [24,25]. In this study, we compared Knosp grade III and IV tumors. Grade IV tumors exhibited firmer consistency, lower GTR rates, poorer postoperative hormonal remission, higher recurrence rates, and a higher incidence of postoperative intracranial infection. Due to complete encasement of the internal carotid artery and firmer texture, grade IV tumors significantly increase surgical difficulty and intraoperative blood loss, contributing to worse prognosis. We conclude that although both grades demonstrate invasive growth, grade IV tumors have a significantly worse prognosis than grade III.

### Limitations

As a retrospective study, the present investigation has two main limitations. First, all data were derived from a single center, which may limit the generalizability of the findings; future research should therefore aim to expand the sample size and incorporate multi-center cohorts. Second, our laboratory testing system only reported a qualitative result of GH > 40 ng/mL (without providing specific quantitative values) when serum GH levels exceeded this threshold. Consequently, preoperative GH levels were categorized as an ordinal variable for subsequent statistical analyses. Third, the WHO 2017 classification first standardized granule subtypes of GH adenomas and identified the sparsely granulated variant as high-risk [41]. The 2022 WHO classification further refined the diagnostic criteria [42]. Following these guidelines, our institution integrated this subtyping into routine pathological analysis. As our cohort spanned 2018–2025, some early pathological records were incomplete; thus, granule subtype was not included in the analysis. Fourth, the follow-up duration of this cohort varied widely, with several patients having a relatively short follow-up and insufficient homogeneity. Further prospective studies with unified standardized follow-up protocols will be needed in the future to validate our results. Fifth, preoperative and postoperative medical therapies were not standardized, and some detailed medication data were incomplete. Thus, the prognostic impact of medical treatment was not analyzed, which warrants investigation in future prospective studies.

## 5. Conclusions

Analysis of risk factors for postoperative hormonal non-remission, tumor recurrence, and persistent hormonal non-remission despite achieving GTR in GHPA patients in this study revealed that those with recurrent disease and higher Knosp grades (especially Knosp grade IV) had significantly worse prognoses. A comparative analysis between high-risk (Knosp grade IV or recurrent cases) and low-risk clinical cohorts demonstrated that recurrent cases exhibited greater tumor invasiveness, firmer tumor consistency, and poorer prognosis compared to primary cases, and Knosp grade IV tumors presented with a firmer consistency and higher surgical resection difficulty than grade III tumors. We constructed nomograms capable of predicting hormonal non-remission as well as the risk of 3-year/5-year tumor recurrence, providing quantitative references for individualized clinical management and follow-up decision-making. Future multicenter studies are still required to further validate and refine the generalizability and clinical utility of the model.

## Figures and Tables

**Table 1:** Analysis of risk factors for postoperative hormonal non-remission (N = 130).

Variable	Univariate Logistic Regression				Multiple Logistic Regression			
	OR	95% CI		*p*	OR	95% CI		*p*
		Lower	Upper			Lower	Upper	
Sex (male)	0.756	0.486	1.175	0.213				
Age	0.972	0.955	0.99	**0.002**	0.988	0.966	1.011	0.294
BMI	0.964	0.911	1.02	0.206				
Hypertension	0.764	0.439	1.33	0.341				
Diabetes	1.158	0.665	2.016	0.604				
Recurrent cases	4.165	2.346	7.397	**<0.001**	2.581	1.267	5.26	**0.009**
Headache	1.554	0.964	2.506	0.070				
Vision defect	1.225	0.763	1.967	0.401				
GH (20–40 ng/mL)	1.676	0.908	3.092	0.099	1.638	0.769	3.488	0.201
GH (>40 ng/mL)	2.609	1.498	4.543	**0.001**	2.133	1.023	4.447	**0.043**
IGF-1/ULN	0.816	0.614	1.085	0.162				
Maximum diameter (mm)	1.055	1.032	1.078	**<0.001**	0.99	0.955	1.025	0.560
Knosp (I)	0.551	0.216	1.404	0.212	0.409	0.14	1.192	0.101
Knosp (II)	0.83	0.345	1.999	0.678	0.719	0.266	1.942	0.516
Knosp (III)	2.434	1.085	5.462	**0.031**	1.967	0.74	5.228	0.175
Knosp (IV)	6.366	2.919	13.882	**<0.001**	4.677	1.651	13.248	**0.004**
Pituitary apoplexy	0.421	0.137	1.298	0.132				
Pituitary cyst	0.648	0.199	2.109	0.471				
Suprasellar	1.338	0.863	2.076	0.193				
GTR	0.09	0.044	0.181	**<0.001**	0.1	0.041	0.247	**<0.001**
Hypervascular	1.348	0.85	2.139	0.204				
Blood loss (mL)	1.001	1	1.001	**0.032**	0.999	0.998	1	0.110
Consistency (Intermediate)	2.395	1.252	4.582	**0.008**	1.739	0.756	3.999	0.193
Consistency (Tenacity)	1.729	1.009	2.963	**0.046**	0.6	0.278	1.294	0.193
Intraoperative CSF Leak	1.315	0.816	2.117	0.260				

All bold values in the table indicate *p* < 0.05.

**Table 2:** Analysis of risk factors for postoperative tumor recurrence (N = 35).

Variable	Univariate COX Regression				Multiple COX Regression			
	HR	95%CI		*p*	HR	95%CI		*p*
		Lower	Upper			Lower	Upper	
Sex (male)	1.141	0.588	2.214	0.697				
Age	0.978	0.952	1.006	0.099				
BMI	0.991	0.909	1.080	0.832				
Hypertension	1.494	0.699	3.188	0.300				
Diabetes	0.907	0.377	2.187	0.828				
Recurrent cases	2.677	1.348	5.318	**0.005**	1.45	0.645	3.260	0.368
Headache	1.101	0.513	2.364	0.806				
Vision defect	2.965	1.526	5.759	**0.001**	2.61	1.253	5.432	**0.010**
GH (20–40 ng/mL)	1.227	0.407	3.700	0.716	1.089	0.357	3.325	0.881
GH (>40 ng/mL)	4.022	1.986	8.150	**<0.001**	3.697	1.635	8.350	**0.002**
IGF-1/ULN	0.994	0.668	1.480	0.975				
Maximum diameter (mm)	1.044	1.022	1.067	**<0.001**	0.972	0.935	1.008	0.138
Knosp (I)	3.329	0.372	29.790	0.282	3.817	0.413	35.280	0.238
Knosp (II)	4.167	0.464	37.439	0.202	4.088	0.443	37.754	0.214
Knosp (III)	5.308	0.617	45.670	0.128	2.931	0.318	26.995	0.343
Knosp (IV)	16.764	2.239	125.483	**0.006**	11.061	1.344	90.938	**0.025**
Pituitary apoplexy	1.593	0.488	5.204	0.441				
Pituitary cyst	0.773	0.106	5.645	0.800				
Suprasellar	1.215	0.621	2.376	0.568				
GTR	0.306	0.154	0.610	**0.001**	0.9	0.373	2.174	0.814
Hypervascular	1.055	0.531	2.094	0.879				
Blood loss (mL)	1.000	1.000	1.000	0.285				
Consistency (Intermediate)	1.518	0.609	3.785	0.370				
Consistency (Tenacity)	1.481	0.673	3.254	0.328				
Intraoperative CSF Leak	0.86	0.403	1.836	0.697				
Ki-67 ≥ 3	2.388	1.227	4.653	**0.011**	0.899	0.400	2.020	0.796
Hormonal non-remission	4.693	2.251	9.787	**<0.001**	2.683	1.062	6.779	**0.037**
LOS	1.085	1.019	1.156	**0.011**	1.047	0.974	1.126	0.220
Postoperative CNS infection	1.628	0.675	3.924	0.278				

All bold values in the table indicate *p* < 0.05.

**Table 3:** Analysis of risk factors for Post-GTR hormonal non-remission (N = 81).

Variable	Univariate Logistic Regression				Multiple Logistic Regression			
	OR	95%CI		*p*	OR	95%CI		*p*
		Lower	Upper			Lower	Upper	
Sex (male)	0.76	0.452	1.278	0.301				
Age	0.981	0.96	1.002	0.069				
BMI	0.961	0.9	1.026	0.235				
Hypertension	0.755	0.388	1.469	0.408				
Diabetes	1.375	0.736	2.57	0.318				
Recurrent cases	3.15	1.555	6.38	**0.001**	2.52	1.188	5.348	**0.016**
Headache	1.409	0.801	2.477	0.234				
Vision defect	0.68	0.367	1.26	0.221				
GH (20–40 ng/mL)	1.308	0.63	2.715	0.471				
GH (>40 ng/mL)	1.789	0.927	3.45	0.083				
IGF-1/ULN	0.832	0.6	1.153	0.269				
Maximum diameter (mm)	1.028	1.002	1.054	**0.033**	0.988	0.956	1.021	0.463
Knosp (I)	0.346	0.109	1.098	0.072	0.403	0.124	1.31	0.131
Knosp (II)	0.936	0.37	2.37	0.889	0.944	0.353	2.526	0.909
Knosp (III)	2.366	0.985	5.682	0.054	2.671	1.005	7.101	**0.049**
Knosp (IV)	3.6	1.517	8.544	**0.004**	4.099	1.418	11.853	**0.009**
Pituitary apoplexy	0.317	0.071	1.42	0.133				
Pituitary cyst	0.742	0.199	2.77	0.657				
Suprasellar	0.976	0.583	1.633	0.926				
Hypervascular	1.348	0.781	2.325	0.283				
Blood loss (mL)	1	0.999	1.001	0.699				
Consistency (Intermediate)	1.48	0.681	3.216	0.322				
Consistency (Tenacity)	0.634	0.287	1.4	0.260				
Intraoperative CSF Leak	1.133	0.642	2.001	0.667				

All bold values in the table indicate *p* < 0.05.

**Table 4:** Comparisons between primary and recurrent cases and between Knosp grade III and IV.

Variable	Primary (N = 280)	Recurrent (N = 64)	*p*	Knosp III (N = 68)	Knosp IV (N = 95)	*p*
Sex			0.609			0.839
Male	128 (45.71%)	27 (42.19%)		29 (42.65%)	39 (41.05%)	
Female	152 (54.29%)	37 (57.81%)		39 (57.35%)	56 (58.95%)	
BMI	26.0 ± 3.9	25.8 ± 4.0	0.748	25.7 ± 3.6	26.3 ± 4.0	0.356
Age	42.6 ± 12.6	38.3 ± 12.6	**0.015**	40.7 ± 12.7	39.1 ± 13.0	0.445
Hypertension	58 (20.71%)	12 (18.75%)	0.725	9 (13.24%)	24 (25.26%)	0.060
Diabetes	53 (18.93%)	11 (17.19%)	0.747	10 (14.71%)	12 (12.63%)	0.702
Headache	81 (28.93%)	16(25%)	0.529	26 (38.24%)	34 (35.79%)	0.750
Vision Defect	73 (26.07%)	29 (45.31%)	**0.002**	24 (35.29%)	37 (38.95%)	0.635
Recurrent case				12 (17.65%)	29 (30.53%)	0.062
IGF-1/ULN	1.9 ± 0.8	1.7 ± 0.6	0.079	1.7 ± 0.7	1.7 ± 0.6	0.830
GH			0.904			0.142
<20 ng/mL	180 (64.29%)	43 (67.19%)		31 (45.59%)	55 (57.89%)	
20–40 ng/mL	44 (15.71%)	9 (14.06%)		17 (25%)	13 (13.68%)	
>40 ng/mL	56 (20.00%)	12 (18.75%)		20 (29.41%)	27 (28.42%)	
Maximum diameter (mm)	20.9 ± 10.6	27.5 ± 12.7	**0.016**	24.3 ± 9.0	31.6 ± 12.3	**<0.001**
Knosp			**0.002**			
0	43 (15.36%)	6 (9.38%)				
I	62 (22.14%)	4 (6.25%)				
II	53 (18.93%)	13 (20.31%)				
III	56 (20.00%)	12 (18.75%)				
IV	66 (23.57%)	29 (45.31%)				
Pituitary apoplexy	16 (5.71%)	3 (4.69%)	0.983	6 (8.82%)	3 (3.16%)	0.225
Pituitary cyst	10 (3.57%)	4 (6.25%)	0.53	4 (5.88%)	3 (3.16%)	0.650
Suprasellar	145(51.79%)	38 (59.38%)	0.272	38 (55.88%)	65 (68.42%)	0.102
Intra-op CSF Leak	78 (27.86%)	21 (32.81%)	0.43	19 (27.94%)	37 (38.95%)	0.145
Hypervascular	182 (65.00%)	39 (60.94%)	0.541	45 (66.18%)	75 (78.95%)	0.068
GTR	247 (88.21%)	37 (57.81%)	**<0.001**	58 (85.29%)	60 (63.16%)	**0.002**
Blood Loss (mL)	243.9 ± 342.5	351.6 ± 337.8	**0.024**	215.3 ± 202.2	433.1 ± 448.2	**<0.001**
Consistency			**<0.001**			**0.027**
Softness	199 (71.07%)	27 (42.19%)		46 (67.65%)	49 (51.58%)	
Intermediate	33 (11.79%)	12 (18.75%)		5 (7.35%)	21 (22.11%)	
Tenacity	48 (17.14%)	25 (39.06%)		17 (25%)	25 (26.31%)	
ki-67 ≥ 3%	87 (31.07%)	37 (57.81%)	**<0.001**	32 (47.06%)	53 (55.79%)	0.271
GH remission	192 (68.57%)	22 (34.38%)	**<0.001**	38 (55.88%)	31 (32.63%)	**0.003**
Post-op Infection	29 (10.36%)	12 (18.75%)	0.062	5 (7.35%)	21 (22.11%)	**0.011**
Tumor recurrence	22 (7.86%)	13 (20.31%)	**0.003**	5 (7.35%)	21 (22.11%)	**0.011**

All bold values in the table indicate *p* < 0.05.

## Data Availability

Data is provided within the manuscript or Supplementary Information Files.

## Supplementary Materials

The following supporting information can be downloaded at https://www.mdpi.com/article/10.3390/curroncol33060310/s1: Table S1: Clinical characteristics of all GHPA patients.
