# Supplementary material for: Clinical Features and Key Prognostic Indicators of Growth Hormone-Secreting Pituitary Adenomas: A Retrospective Study of 344 Cases
Source: Curr Oncol. 2026 May 27;33(6):310. doi: 10.3390/curroncol33060310 (PMC13298231; doi:10.3390/curroncol33060310)
Supplement: Supplementary file 1 [file curroncol-33-00310-s001.zip › curroncol-4256592-supplementary.pdf]

Table S1 Clinical characteristics of all GHPA patients

Variable	N=344（%）
**General Information**	
Sex	
Male	155（45.1）
Female	189（54.9）
Age	41.76±12.67
BMI	25.94±3.90
**Pre-op Information**	
Hypertension	70（20.3）
Diabetes	64（18.6）
Recurrent cases	64（18.6）
Primary cases	280（81.4）
Headache	97（28.2）
Vision defect	102（29.7）
GH	
<20ng/ml	223(64.8)
20-40ng/ml	53(15.4)
>40ng/ml	68(19.8)
IGF-1/ULN	1.87±0.80
**Imaging Assessment**	
Maximum diameter（mm）	22.13±11.30
Knosp	
0	49(14.2)
I	66(19.2)
II	66(19.2)
III	68(19.8)
IV	95(27.6)
Pituitary apoplexy	19(5.5)
Pituitary cyst	14(4.1)
Suprasellar	183(53.2)
**Intra-op Information**	
GTR	284(82.6)
Hypervascular	221(64.2)
Blood loss（ml）（20-3500）	263.95±343.72
Consistency	
Softness	226（65.7）
Intermediate	45（13.1）
Tenacity	73（21.2）
Intraoperative CSF Leak	99（28.8）
**Post-op Information**	
Ki-67≥3	124（36.0）
Hormonal remission	214（62.2）

Hormonal non- remission	130 (37.8)
LOS	9.44±3.82
Postoperative CNS infection	41（11.9）
Postoperative Recurrence	35（10.2）
